# Job demands-resources, job crafting and work engagement of tobacco retailers

**DOI:** 10.3389/fpubh.2022.925668

**Published:** 2022-08-22

**Authors:** Daokui Jiang, Lei Ning, Teng Liu, Yiting Zhang, Qian Liu

**Affiliations:** ^1^Business School, Shandong Normal University, Jinan, China; ^2^Economics and Management School, Guangdong Construction Polytechnic, Guangzhou, China

**Keywords:** job resources, job demands, job crafting, work engagement, servant leadership

## Abstract

In recent years, the development of tobacco control actions in China and the changes in people's health concepts have slowed the development of the tobacco industry. As an important strategic partner of tobacco sales companies, tobacco retailers are the key link between tobacco commercial enterprises and consumers. How to improve the work engagement level of tobacco retailers is an urgent issue for tobacco business enterprises. On the basis of job demands–resources (JD–R) theory, the mechanisms of the effects of job resources and demands on tobacco retailers' work engagement were explored. Results showed that (1) The negative path of job demands influencing tobacco retailers' work engagement was supported, and job crafting played a mediating role in it. (2) The positive path of job resources influencing tobacco retailers' work engagement was supported, and job crafting played a mediating role in it. (3) Servant leadership moderated the influence of job resources and demands on job crafting. Higher level of servant leadership brings the stronger effect of job resources on job crafting and the weaker effect of job demands on job crafting. (4) The mediating effect of job crafting between JD–R and work engagement was moderated by servant leadership. The higher level of servant leadership strengthened the mediating role of job crafting between job demands and work engagement, whereas it weakened the mediating role of job crafting between job demands and work engagement. This study enriches the research on application fields and boundary conditions of JD–R theory and provides practical guidance for improving the work engagement level of tobacco retailers.

## Introduction

Work engagement, referring to the positive behavior or positive attitude of individuals in their work, has always been a topic that scholars have focused on. Individuals with high work engagement tend to be energetic, more focused on their work, and willing to contribute to the organization (1). They are more likely to be satisfied and happy at work, which has a positive effect on team performance. Therefore, many enterprises and scholars are committed to exploring the mechanism of maintaining individuals' high work engagement, aiming to reduce “loafing on the job” of employees in the “management blind area” and then improve the overall efficiency of the organization (2, 3). However, the work engagement of formal employees in the enterprise has received attention, whereas that of the informal employees affiliated to the company have been ignored. In fact, the level of work engagement of informal employees is highly important to the organizational performance of the company to which the employee belongs, which is the case with tobacco retailers.

As an important strategic partner of tobacco sales enterprises, tobacco retailers are the key link between tobacco commercial enterprises and consumers. Given the special influence of the monopoly system of the tobacco industry, tobacco retailers are not viewed as regular employees of tobacco commercial enterprises, but their business behavior is managed and restricted by the tobacco industry system. Notably, the business performance of these informal employees directly influence enterprise performance. Tobacco retailers, tending to be far away from the direct supervision of enterprises, have a certain autonomy in business concept and behavior, and their work form is also more flexible. However, a high degree of self-management may influence the level of work engagement and the retail performance of tobacco retailers. The work engagement level of tobacco retailers not only affects the business performance and consumer experience but also the brand cultivation of the tobacco industry. Therefore, effectively improving the work engagement level of tobacco retailers plays an important role in the high-quality development of tobacco commercial enterprises.

Previous study indicated that matching reasonable demands and resources for employees is the key to improving employees' work engagement (4). Job demands–resources (JD–R) theory proposes that any job characteristic can be divided into job demands and resources. Job demands cause increase in job stress, employees' job burnout, and the loss of individual physical and mental health; whereas job resources can improve work engagement and promote employees' growth (5).

Tobacco retailers are the special group of tobacco commercial enterprises. On the one hand, their retail business is subject to the jurisdiction and guidance of the tobacco company. On the other hand, they have the right to operate and manage their stores independently. Such special job characteristics bring them job demands and resources. They need to be trained by tobacco companies and adjust to the transformation of tobacco business model, which may greatly increase their workload and require higher standards on their job skills. These job demands may reduce their work engagement. However, they also enjoy work autonomy and have some spaces to make their own decisions at work, such as selecting cigarette categories. They can also obtain guidance and help from tobacco account managers (this position is mainly set up to serve tobacco retailers) at any time in business difficulties. This type of work support has a positive effect on work engagement. Therefore, retailers' work engagement may be affected by job demands and resources. The JD–R model provides a theoretical basis for exploring this impact mechanism. The JD–R model can simultaneously analyze job demand and job resource elements under one framework and organically combine the positive and negative effects of job characteristics on work engagement to form a balanced, comprehensive, and interactive analysis framework.

Self-regulation theory holds that when the external environment changes, individuals have the ability to self-adjust and motivate themselves, and can actively adjust their attitudes and behaviors. Therefore, when job demands and resources change, employees usually take the initiative to make positive responses to coordinate the status quo of demands and resources and better adapt to work. These proactive responses often manifest as job crafting behaviors. Research shows that employees' active transformation of work will bring many positive results, such as work engagement and job satisfaction (6). Therefore, job crafting may play an intermediary role between JD–R and work engagement. In addition, as employees' important guides in the organization, leaders may influence subordinates' work attitude and behavior considerably, and this influence is mainly reflected through leadership (7). Servant leadership attaches importance to the interests of their followers and are willing to give up their own interests and serve others (8). Thus, wining the trust of subordinates is easy for them, and subordinates do not worry about the consequences of failure, resulting in stronger motivation for job crafting. Therefore, servant leadership style may moderate the effects of job characteristics on job crafting.

This study aims to provide practical guidance for improving the level of work engagement of tobacco retailers by adopting the widely recognized and mature theory of JD–R and extending its model to tobacco retailers, and these research results can help with the management of informal employees that exists in other industries. This study has three main contributions. First, we pay attention to the special group of tobacco retailers and apply the JD–R model to explore their work engagement, thereby expanding the application scope of JD–R model. Second, this study constructs the impact path model of job demands and resources on work engagement, which enriches the theoretical results of JD–R theory. Third, in view of the indispensable role of leadership style in the influence of JD–R on work engagement, we take servant leadership as a moderate variable to reveal the internal mechanism and boundary conditions between JD–R and work engagement.

This study focuses on a major research gap in which previous research on employee work engagement has adopted a decentralized approach. To be specific, most studies have only explored the effect of a specific job characteristic factor on work engagement based on the explanatory perspective of a single independent variable and a mediating variable. However, these studies have failed to provide a comprehensive, broad, and flexible model of work engagement for tobacco retailers. Empirical studies are needed to investigate work engagement at tobacco retailers. In this study, the theoretical framework of JD–R was adopted, and the combination of job resources and demands into the analytical framework would ultimately help improve tobacco retailers' work engagement.

This paper begins with an introduction to JD–R theory, which guides this study and contributes to the formulation of the hypotheses to be tested. Subsequently, we describe the overview of the analysis strategy, data collection, variable measurement, and data analysis to test the hypotheses. Finally, this study is concluded with discussion of the results, implications, limitations, and future research avenues.

## Literature review and hypotheses

### JD–R theory

JD–R theory was first proposed by Demerouti et al. (9) to explain the relationship between job characteristics and individual perception factors, such as job burnout. Its core connotation is that any job characteristic can be divided into two types: job demands and resources. Job demands are the factors that drain individual energy and require individual efforts or costs to complete the work, such as job overload, role conflict, time pressure, and job insecurity. On the contrary, job resources are positive factors in work, which refer to relevant factors in work that can promote the realization of work objectives and help individuals grow, learn, and develop. The theory mainly studies the interaction between work environment and individual work performance and reflects the dynamic mechanism of “job characteristics and work performance” through the changes of job demands and resources (9).

On the basis of the core connotation, JD–R theory develops three core hypotheses. The first is the “dual path” hypothesis, that is, there exist two influence paths of job characteristics on employees' loss and gain. Excessive job demands will continue to drain employees' energy, which will have a negative effect on their work results. On the contrary, sufficient job resources can trigger employees' initiative to improve the level of work engagement and have a positive effect on their work results. The second is the buffer hypothesis, which holds that job resources can buffer the loss of high level of job demands on employees and reduce the negative effects of job demands on them. The third is the cope hypothesis, which holds that employees can make better use of job resources to complete work objectives under a high level of job demands.

The JD-R model has been tested in teachers, health care workers and doctors in Finland, the Netherlands and Germany, making great breakthroughs in theory and practice. Some scholars carried out studies based on samples from different countries (regions) and different occupations, and more consistently confirmed the hypothesis in JD-R model that there is a positive correlation between demands and negative emotional perception (such as job burnout), or a negative correlation between job demands and positive emotional perception (such as job satisfaction). The results consistently support the gain path that job resources can positively influence positive emotions (10–12).

On the basis of the core connotation of JD–R theory and its dual path hypothesis, this paper explores the impact mechanism of JD–R on the work engagement of tobacco retailers while considering the influence of servant leadership on this process.

### JD–R and job crafting

Job demands refer to the efforts and costs needed to complete the work, including work intensity, work ability requirements, and work family conflicts, which constitute the negative factors that consume individual energy at work and form a loss path for individuals from work (13). Most of tobacco retailers have low educational level, and they often participate in training to improve their work skills. The tobacco commercial companies implement assessment and ranking of their business performance, which brings them psychological pressure. In recent years, the tobacco business model is undergoing digital transformation, and tobacco retailers need to pay more working time and workload to complete their work in the process of transformation, which increases their job requirements. Existing studies have indicated that job demands can lead to employees' negative psychological and behavioral feedback, thereby reducing the level of work engagement (14). Excessive job demands will make employees have a bad perception of the working environment. If this process lasts for a long time, it will force employees to invest more energy in work and consume employees' physical and psychological resources (15). On the basis of resource conservation theory, individual resources are limited. When individuals perceive the loss of resources, they tend to take actions to protect and maintain existing resources and seek new ones. When the work consumes too much personal resources, employees will no longer be able to calmly deal with the work, resulting in job burnout (16). Then employees will take a negative attitude and behavior toward the work, and they cannot maintain energy at work, which will affect employees' work enthusiasm to a great extent.

Job crafting refers to a series of proactive behaviors that employees show to balance job resources and demands. High job demands lead to psychological changes of employees (17). When job responsibilities exceed job resources, employees will feel great work pressure, resulting in adverse emotions, such as anxiety and pain, and difficulty in further devoting themselves to work. For the group of tobacco retailers, in addition to physical effort at work, emotional labor is also required. This mental cost can lead to emotional exhaustion, which can lead to negative emotions, such as depression, anxiety, and burnout. As a result, excessive work demands exert psychological and physical pressure on tobacco retailers. Then, they would respond negatively to their work rather than actively take measures to change the current situation. On the basis of the views above, we propose the following hypothesis:

*H1a: Job demands have negative effects on employees' job crafting behavior*.

Job resources refer to physical, psychological, social and organizational factors at work. These factors, including key resources, such as work autonomy, social support, and performance feedback, usually play a role in reducing psychological consumption, achieving work goals, and promoting personal development (18). Tobacco retailers have autonomy in their work. They can choose the category of cigarettes they sell and independently determine the display style of the store. At the same time, these decisions and other work will receive professional guidance and timely feedback from the tobacco customer manager. Account manager is a position set up by the tobacco commercial companies to serve their retailers, which can support the retailers' work and help them solve the problems in their work. Research shows that job resources have direct and indirect effects on employee engagement, emotional commitment, and turnover intention (19). Job resources can stimulate employees' motivation and make them feel that work is more meaningful, so they are more responsible for the work process and work results and are more active in work (10). Employees with sufficient job resources can experience more autonomy in their work (20), have a higher sense of control over their work, and can use resources to reshape their work according to their own needs. When individuals perceive the lack of resources and cannot respond to job demands, job burnout will occur. On the contrary, if the organization provides employees with more job resources, such as more support and work autonomy, which can make employees maintain positive emotions at work, then job crafting behavior will occur. Work autonomy, work support from customer managers, and work feedback increase the job resources of tobacco retailers, which can stimulate their motivation to actively improve their work and promote job crafting behavior. On the basis of the views above, we propose the following hypothesis:

*H1b: Job resources have positive effects on employees' job crafting behavior*.

### Mediating role of job crafting

Job crafting is the initiative of employees to meet individual or organizational needs and make their work more meaningful. Tims et al. (21, 22) based on JD–R theory, defined job crafting as the changes taken by individuals to achieve balance between job demands and resources. As a spontaneous behavior of employees, job crafting is conducive to individuals' obtaining job meaning and reconstructing employee identity (23). Previous studies have shown that job crafting can significantly promote work status and results. Therefore, job crafting is considered to have a positive effect on employees' motivation, dedication, commitment, and work engagement (24, 25).

On the one hand, employees can improve their ability and obtain a sense of achievement by participating in more challenging work tasks. On the other hand, employees can obtain job feedback and better respond to job requirements by communicating with more colleagues. Therefore, in this process, employees' competence and interpersonal relationships are improved. When the internal needs of employees are realized, their investment level will also be improved. Many tobacco retailers have reinvented their work. For example, they take the initiative to participate in various skills training to improve their operating capacity and actively participate in mutual assistance activities of integrity groups. These job crafting behaviors can increase turnover, align retailers' individual value goals with the tobacco company's goals and consumer interests, and then demonstrate better work engagement levels in the workplace. On the basis of above, we propose the following hypothesis:

*H2: Job crafting has a positive influence on employees' work engagement*.

On the basis of JD–R theory, Tims et al. (21, 22) defined job crafting as “the change in behavior that employees actively make in order to adapt their work ability to job demands.” Employees' job crafting behavior is mainly to increase job resources or reduce job demands. Therefore, job crafting is a strategy for individuals to optimize their work characteristics, which can help individual characteristics adapt to job demands to increase work engagement (26).

According to the self-regulation theory, when the current state is inconsistent with the expected state, the individuals will awaken the self-regulation mechanism, that is, individuals will actively take action to change the current state to achieve the expected state. Therefore, when the job demands and resources change, individuals may not passively accept it, but they will take the initiatives to improve their adaptability (27). From the perspective of tobacco retailers, when they are faced with high-level job demands, considering their generally low education level and high age, they are less likely to overcome difficulties and seek self-renewal, and there exist more obstacles to conduct job crafting. However, when they have more job resources in their work, such as the careful guidance and work affirmation form customer managers, their motivation to take the initiative to change themselves becomes stronger, and taking action of job crafting becomes more possible. Through job crafting, tobacco retailers can effectively prevent the loss of job resources and the increase of job demands. Employees meet the needs of autonomy through job crafting, are full of vitality at work, and improve the level of work engagement.

To sum up, when job resources and demands change, employees can reshape their behavior through job crafting, make themselves better fit their job, and increase their work engagement level. On the basis of the views above, we propose the following hypotheses:

*H3a: Job crafting mediates the relationship between job demands and work engagement*.

*H3b: Job crafting mediates the relationship between job resources and work engagement*.

### Moderating role of servant leadership

As the initiative behavior of individual employees, job crafting is affected by the external environment, such as organizational atmosphere and leadership style (28). Servant leadership is a leadership style that is willing to serve employees and put employees' emotions and needs in an important position. Thus, servant leadership plays a positive role in promoting positive work behaviors, such as job crafting and innovative work behavior (29). Tobacco account managers can be regarded as servant leaders of retailers. They help manage the store operations of retailers, guide their work, pay attention to their growth, and are willing to provide services for their work, which are of great significance to improve the work engagement of retailers.

As an emerging leadership style, servant leadership will affect employees' work behavior (30). On the one hand, employees' job crafting not only can promote the realization of work goals but also meet personal needs (31). In the work process, employees feel the organizational atmosphere through their leadership style to judge whether their behavior can be recognized by the organization and determine the follow-up work and job crafting behavior. On the basis of the principle of altruism, servant leadership style show the characteristics of serving subordinates and safeguarding their rights and interests in their work (32), convey the signal that the organization attaches importance to employees, and promote employees to actively solve problems in their work. On the other hand, servant leadership leaders give employees work autonomy through authorization, alleviate resource loss, and encourage employees to take positive ways to deal with changes in work characteristics. Therefore, servant leadership leaders pay attention to the satisfaction of employees' growth needs, improve employees' sense of identity, enhance employees' sense of belonging (8), and improve employees' work engagement level (33). On the basis of the views above, we propose the following hypotheses:

*H4a: Servant leadership positively moderates the effects of job resources on job crafting. Specifically, the higher (lower) the level of servant leadership perceived by employees is, the stronger (weaker) the positive effects of job resources on job crafting will be*.

*H4b: Servant leadership negatively moderates the effects of job demands on job crafting. Specifically, the higher (lower) the level of servant leadership perceived by employees is, the weaker (stronger) the negative effects of job demands on job crafting will be*.

This paper holds that servant leadership plays a regulatory role among job resources, job demands, and work engagement. Servant leadership can help individuals set the path to achieve goals, provide support, and remove obstacles for the final achievement of goals and actively respond to the needs of employees in their work. On the basis of social exchange theory, when employees perceive the support behavior of servant leadership, under their subtle guidance and support, they are more likely to have a sincere attitude of gratitude to the organization, continuously produce value to meet the requirements of organizational development, and improve the level of work engagement (33). For retailers who acquire the service and guidance of customer managers in time, they can feel that they are valued and have sufficient resources to improve their business ability to stimulate their enthusiasm for continuous improvement and improve their work engagement. However, retailers without such servant leadership resources are more likely to withdraw and reduce work engagement when encountering work obstacles.

On the basis of the above analysis, servant leadership moderates the relationship among job resources, job demands, and work engagement. At the same time, based on *H3a, H3b, H4a*, and *H4b*, we believe that servant leadership moderates the mediating role of job crafting among job resources, job demands, and work engagement by moderating the relationship among job resources, job demands, and job crafting. On the basis of the views above, we propose the following hypotheses:

*H5a: The mediating effect of job crafting between job resources and work engagement is moderated by servant leadership. Specifically, the higher (lower) the level of servant leadership is, the stronger (weaker) the mediating effect will be*.

*H5b: The mediating effect of job crafting between job demands and work engagement is moderated by servant leadership. Specifically, the higher (lower) the level of servant leadership is, the weaker (stronger) the mediating effect will be*.

**Figure 1:**
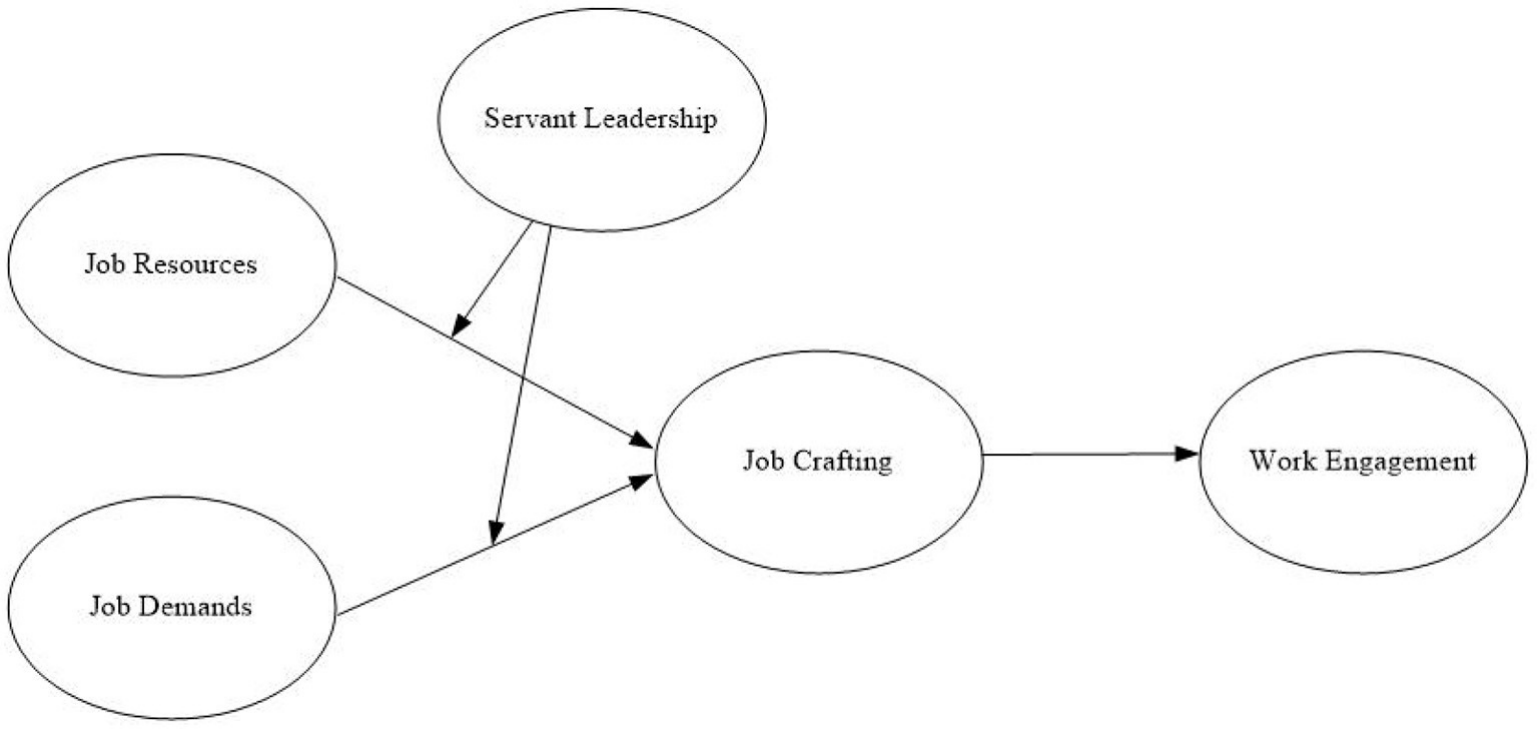
Research model.

The research model is shown in Figure 1.

## Materials and methods

### Sample

This study took tobacco retailers as the research object and conducted research in Shandong Jining Tobacco Company. The whole data collection process is divided into two steps. First, before the formal investigation, two experienced researchers go to the target enterprise to communicate with the enterprise leaders on the spot. The researcher explained the purpose of the study and the potential benefits for the company to the company representative and asked the account manager to encourage the retailers to actively participate in the survey within the work group. To ease the concerns of the respondents, we emphasized anonymous submission of questionnaires and strict confidentiality of data. Second, online questionnaires were distributed by the direct superior leaders (account managers) of tobacco retailers during their work. During the questionnaire filling period, the researcher would keep in touch with the respondents and answer their questions in time to gain their trust. In addition, to improve the validity and authenticity of the data, a lie detection question was set in the questionnaire to check whether the respondents answered the questions carefully. In addition, to improve the enthusiasm of the subjects, a red envelope of 1–5 reward was randomly attached at the end of each questionnaire. A total of 500 questionnaires were distributed, and 470 questionnaires were recovered. After excluding the questionnaires with incomplete filling and obvious logical errors, 437 valid questionnaires were finally obtained, and the recovery rate of valid questionnaires was 87.4%. Particularly, 41.4% were male, and 58.6% were female; 44.2% were aged 35 and below, 35.7% were aged 36–45, and 20.1% were aged 46 and above; 45.7% had a high school degree or below, 9.2% had a college degree, and 45.1% had a bachelor's degree or above.

### Variables

All of the measurement scales were established well and drawn from the literature. The survey was administered in Chinese. All items used the same seven-point Likert scale format (1 = strongly disagree, 7 = strongly agree).

#### Job resources

Job resources was measured using a six-item scale developed by Demerouti et al. (9), including “I am passionate about my work” and “I am proud of what I do.” The internal consistency coefficient is 0.905.

#### Job demands

Job demands was measured using an eight-item scale developed by Karasek et al. (34), such as “I never have enough time to finish everything.” The internal consistency coefficient is 0.930.

#### Job crafting

Job crafting was measured using a seven-item scale developed by Petrou et al. (24) and Demerouti and Peeters (35), such as “I look for various ways to improve work efficiency at work.” The internal consistency coefficient is 0.910.

#### Servant leadership

Servant leadership was measured using a seven-item scale developed by Liden et al. (32), such as “My superior attaches great importance to my career development.” The internal consistency coefficient is 0.910.

#### Work engagement

Work engagement was measured using a three-item scale developed by Rothbard (36), including “I focus a lot of attention on my work.” The internal consistency coefficient is 0.890.

## Results

### Reliability and validity measurements

Confirmatory factor analysis was conducted using Amos 22.0 to assess the measurement model and test the reliability and validity of the constructs. The results are shown in Tables 1, 2. As shown in Table 1, first, the standardized factor load of each item in the scale is >0.7, indicating that the scale has a good fitting degree. Second, the composite reliability of the model variables is all >0.70, and the average variance extracted (AVE) is all >0.5, which reach the judgment standard suggested by Hair et al. (37). Thus, the scale has excellent internal consistency.

**Table 1:** Standardized factor loading, construct reliability and convergent validity.

**Constructs**	**Items**	**Standardized factor loading**	***t*-value**	**Composite Reliability (CR)**	**Average Variance Extracted (AVE)**
Job resources	JR1	0.852	40.571^***^	0.913	0.673
	JR2	0.806	23.706^***^		
	JR3	0.705	20.143^***^		
	JR4	0.710	22.188^***^		
	JR5	0.818	30.296^***^		
	JR6	0.882	55.125^***^		
Job demands	JD1	0.744	21.882^***^	0.942	0.671
	JD2	0.744	23.250^***^		
	JD3	0.778	18.093^***^		
	JD4	0.815	23.971^***^		
	JD5	0.910	56.875^***^		
	JD6	0.827	63.615^***^		
	JD7	0.884	42.095^***^		
	JD8	0.834	32.077^***^		
Job crafting	JC1	0.703	19.528^***^	0.911	0.596
	JC2	0.706	20.765^***^		
	JC3	0.845	38.409^***^		
	JC4	0.847	38.500^***^		
	JC5	0.721	16.767^***^		
	JC6	0.776	20.421^***^		
	JC7	0.792	25.548^***^		
Work engagement	WE1	0.743	19.553^***^	0.855	0.665
	WE2	0.820	21.579^***^		
	WE3	0.877	35.080^***^		
Servant leadership	SL1	0.763	26.310^***^	0.932	0.665
	SL2	0.820	32.801^***^		
	SL3	0.889	55.563^***^		
	SL4	0.836	30.963^***^		
	SL5	0.889	38.652^***^		
	SL6	0.719	18.921^***^		
	SL7	0.775	26.724^***^		

*** indicate significance at p < 0.001, respectively.

**Table 2:** Result of CFA.

**Model**	**CMIN**	**Df**	**CMIN/DF**	**RMSEA**	**NFI**	**CFI**	**TLI**
One-factor^a^	4487.158	427	10.509	0.148	0.585	0.607	0.572
Two-factor^b^	3967.225	426	9.313	0.138	0.633	0.658	0.626
Three-factor^c^	2100.667	424	4.978	0.096	0.805	0.837	0.821
Four-factor^d^	1655.390	421	3.932	0.082	0.847	0.881	0.868
Five-factor^e^	975.285	417	2.339	0.055	0.910	0.946	0.940
ULMC^f^	963.527	416	2.316	0.055	0.911	0.947	0.941

= 437. RMSEA, Root Mean Square Error of Approximation; JR, job resources; JD, job demands; JC, job crafting; WE, work engagement; SL, servant leadership.

a One-factor, all variables merged.

b Two-factor, JR, JD + JC + SL + WE.

c Three-factor, JR, JD, JC + SL + WE.

d Four-factor, JR, JD, JC + SL, WE.

e Five-factor, JR, JD, JC, SL, WE.

f ULMC, Five-factor + CMB.

Table 2 shows the model fitting index. By comparing the five-factor measurement model of the five constructs, namely, job resources, job demands, job crafting, work engagement, and servant leadership, with the alternative models, including four, three, two, and single factors, the five-factor model fit is the best (χ2 = 975.285, Df = 417, χ2/df = 2.339, RMSEA = 0.055, NFI = 0.910, CFI = 0.946, TLI = 0.940), showing good discriminative validity.

In addition, the square root of AVE of each variable is greater than the correlation coefficient, indicating good discriminant validity (Table 3).

**Table 3:** Descriptive statistical analysis results.

**Variables**	**Mean**	**SD**	**1**	**2**	**3**	**4**	**5**
1.JR	5.527	1.142	**0.820**				
2.JD	3.685	1.197	−0.198^***^	**0.819**			
3.JC	5.336	1.121	0.548^***^	−0313^***^	**0.772**		
4.WE	5.233	1.092	0.452^***^	−0.113^***^	0.619^***^	**0.815**	
5.SL	5.036	1.209	0.621^***^	−0.312^***^	0577^***^	0.561^***^	**0.816**

*** indicate significance at the level of p < 0.001.

Diagonal bold numbers are the square root of AVE. JR, job resources; JD, job demands; JC, job crafting; WE, work engagement; SL, servant leadership.

### Evaluation of common method bias

To test the CMB, the following methods were adopted in this paper. First, the results of the Harman single-factor test show that all the items were analyzed into five factors, and the first factor accounts for 36.9% of all variance, no more than 40%, indicating that the deviation of the common method is not serious. Second, referring to the treatment of Podsakoff et al. (38) and Liang et al. (39), this study used the unmeasured latent method construct to test the effect of common method variance, and the results are as follows: ΔRMSEA = 0, ΔNFI = 0.001, ΔCFI = 0.001, ΔTLI = 0.001 (Table 2). The changes in all indicators are <0.002, and the model fit does not improve. On the basis of the above judgment, the effect of common method variance is not serious in this study.

### Descriptive statistical analysis

The mean, standard deviation, and correlation coefficient of variables are summarized in Table 1. The correlation coefficients of all variables are below 0.7, so no multicollinearity problem exists. Job resources and demands are significantly correlated with job crafting (r = 0.548, *p* < 0.001; r = −0.313, *p* < 0.001), and job crafting is significantly correlated with work engagement (r = 0.619, *p* < 0.001). Servant leadership is positively correlated with job crafting and work engagement (r = 0.577, *p* < 0.001; r = 0.619, *p* < 0.001). The overall results provide preliminary support for the further test of hypotheses.

### Hypothesis testing

#### Testing the main and mediating effects

In order to test the hypothetical model proposed in this paper, this study used the multilevel structural equation model to compare the theoretical, nested, and alternative models to find the optimal one (40). In the theoretical model, job resources and demands do not have a direct effect on work engagement, whereas the nested model adds a direct effect based on the theoretical model, and no mediation effect exists in the alternative model. That is, job resources, job demands, and job crafting directly affect work engagement. The model fit is shown in Table 4.

**Table 4:** Model fit of the theoretical model, nested model and alternative model.

**Model**	**CMIN**	**DF**	**CMIN/DF**	**RMSEA**	**CFI**	**TLI**	**ΔCMIN (ΔDF)**
M1: theoretical model	734.088	234	3.137	0.070	0.936	0.924	
M2: nested model	733.044	233	3.146	0.070	0.936	0.924	1.044 (1)
M3: alternative model	770.261	231	3.334	0.073	0.931	0.917	36.173 (3)

ΔCMIN (ΔDF), compared with M1.

Table 3 shows that the model fit of the theoretical and nested models are good. According to Anderson and Gerbing (41), the change of the chi-square value of the theoretical model and the nested model was insignificant (ΔCMIN = 1.044, *p* > 0.05), which indicates that adding the direct path did not significantly improve the model fit. The model fit of the alternative model is also good. According to Vrieze (42), by comparing the Bayesian information criterion (BIC), when ΔBIC > 10, the model with a smaller BIC is better. The BIC of the theoretical model is 1135.364, whereas the alternative model is 1193.353, and the ΔBIC is 57.989, indicating that the theoretical model is better than the alternative model. Therefore, the theoretical model can reflect the relationship between variables more effectively than the nested and alternative models.

**Figure 2:**
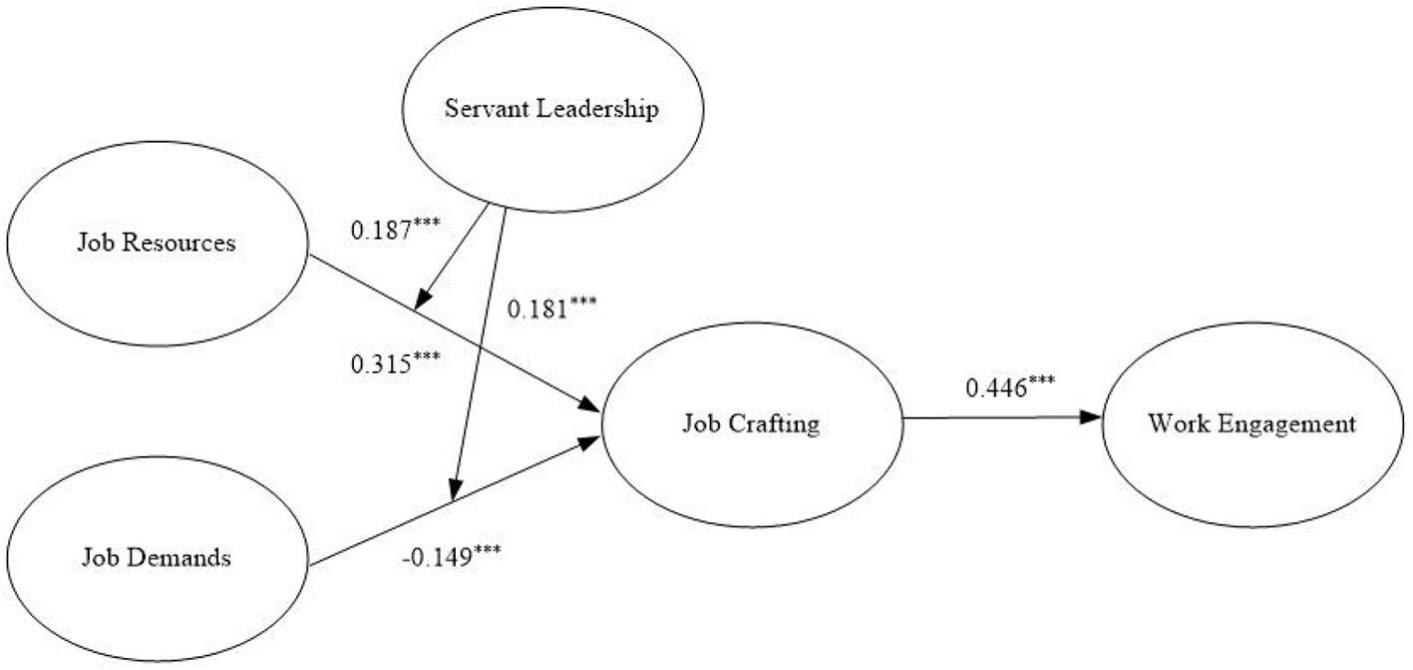
Results of the theoretical model.

Figure 2 shows the results of the theoretical model. After controlling for variables of age, gender, and education level, job resources (b = 0.315, *p* < 0.001) and job demands (b = −0.149, *p* < 0.001) have a significant effect on job crafting, and job crafting has a significant effect on work engagement (b = 0.446, *p* < 0.001). Therefore, H1a, H1b, and H2 are all supported.

The bootstrap method was used to test the mediation effect, and the results are shown in Table 5. Combining Figure 2 and Table 5 indicates that job crafting has a significantly positive effect on work engagement (b = 0.446, *p* < 0.001). The mediating effect of job crafting among job resources (b = 0.140, *p* < 0.001), job demands (b = −0.066, *p* < 0.01), and work engagement is significant, and the confidence intervals does not include 0, [0.153, 0.318] and [−0.173, −0.077]. Therefore, H3a and H3b are supported.

**Table 5:** Bootstrapping test results of mediating effect.

**Effect path**	**Mediate effect**	**95% confidence interval**	
		**Lower limits**	**Upper limits**
JR → JC → WE	0.140^***^	0.153	0.318
JD → JC → WE	−0.066^***^	−0.173	−0.077

*** indicate significance at p < 0.001, respectively.

### Test of moderating effect

Latent moderated structural equations were used to test the moderating effect of servant leadership (Figure 2). The results shows that the interaction between job resources and servant leadership (b = 0.187, *p* < 0.001) and that between job demands and servant leadership (b = 0.181, *p* < 0.001) are significant, indicating that servant leadership moderates the effect of job resources and requirements on job crafting.

**Figure 3:**
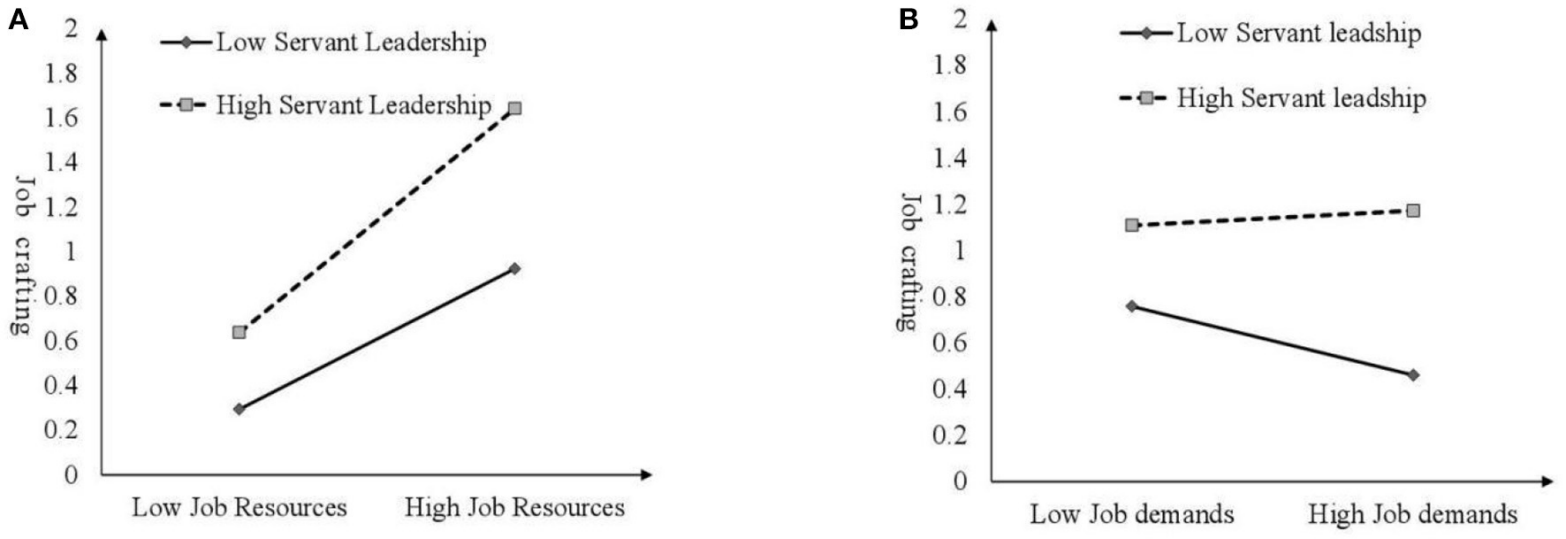
**(A)** The moderating role of servant leadership. **(B)** The moderating role of servant leadership.

To more clearly show the moderation effect of servant leadership, we conducted a simple slope test according to the method recommended by Aiken and West (43), and we drew the moderation effect diagram. As shown in Figure 3A, when the level of servant leadership is high, the positive effect of job resources on job crafting is highly significant. On the contrary, when the level of servant leadership is low, the positive effect of job resources on job crafting is weakened, and there exists a significant difference between high and low levels of servant leadership. Thus, H4a is supported. As shown in Figure 3B, when the level of servant leadership is low, the negative effect of job demands on job crafting is significant. On the contrary, when the level of servant leadership is high, the effects of job demands on job crafting are weakened, and there exists a significant difference between high and low levels of servant leadership. Thus, H4b is supported.

### Test of moderated mediation effect

According to the suggestions of Edwards et al. (44), this paper used a bootstrapping method to analyze the mediating effect of job crafting among job resources, job demands, and work engagement under different servant leadership levels. The analysis results are shown in Table 4.

First, under the high level of servant leadership, the effects of job resources on job crafting are significant (b = 0.379, *p* < 0.001), and the difference between high and low levels of servant leadership is significant (Δb = 0.236, *p* < 0.001), indicating that servant leadership positively moderates this relationship. As shown in Table 6, the indirect effect of job resources on work engagement is significant under a high level of servant leadership (b = 0.158, *p* < 0.001), and the difference between high and low servant leadership level is significant (Δb = 0.100, *p* < 0.001).

**Table 6:** Moderated mediation effect test.

**IV**	**Moderator**	**Path**	**Path**	**Conditional**
	**SL**	**IV → JC**	**JC → WE**	**Indirect Effect**
JR	Low (3.817)	0.144^**^	0.403^***^	0.058^***^
	High (6.235)	0.379^***^	0.417^***^	0.158^***^
	Difference	0.236^***^	0.014^***^	0.100^***^
JD	Low (3.817)	−0.238^***^	0.521^***^	−0.124^*^
	High (6.235)	−0.030	0.533^***^	−0.016^*^
	Difference	−0.108^**^	0.012^***^	−0.108^**^

* ,

** , and

*** , respectively, indicate significance at the level of p < 0.05, p < 0.01, and p < 0.001. Low SL, mean standard deviation; High SL, mean + standard deviation; IV, Independent variable.

Second, under a low level of servant leadership, the effects of job demands on job crafting are significant (b = −0.238, *p* < 0.001), and the difference between high and low levels of servant leadership is significant (Δb = −0.108, *p* < 0.01), indicating that servant leadership negatively moderates this relationship. As shown in Table 6, the indirect effect of job demands on work engagement is significant under a low servant leadership level (b = −0.124, *p* < 0.05), and the difference is significant under high and low levels of servant leadership (Δb = −0.108, *p* < 0.01).

On the basis of the above results, H5a and H5b are supported.

## Discussion

### Conclusion

On the basis of the JD–R model, this paper conducted a theoretical analysis of the influencing factors of job remodeling in tobacco retailers and further investigated how job demands and resources affect the work engagement of tobacco retailers through job crafting. This paper finally draws the following conclusions.

First, job resources positively influence job crafting, and positively influences work engagement through job crafting. The results of our data suggest that tobacco retailers who have access to more job resources show greater motivation to reshape their jobs and have higher levels of work engagement. These results are consistent with the findings of Albrecht et al. (45) and Chen et al. (4), who proposed that adequate work resources can improve employees' creative performance and work engagement level. Knight et al. (46) claimed that the construction of personal and job resources is an effective work engagement, which confirms our conclusion. In addition, job crafting has a positive effect on work engagement, which is consistent with Teng et al. (47).

Second, job demands negatively influence job crafting and negatively affect work engagement through job crafting. Although the JD–R model does not assume any direct link between job demand and work engagement, an ongoing debate exists about the effects of job demands on work engagement (48), partially because whether job demands have positive or negative effects on work engagement is unclear. This study confirms that job demands have negative effects on work engagement, which is consistent with the findings of Oshio et al. (49) and Breevaart and Bakker (50). Tobacco retailers, who are being asked to digitally transform, need to take on more workload than ever to adapt to the new retail model, and those who lack business skills often need to spend more time doing the work. Those job demands lead to a decline in their work engagement level. Third, servant leadership plays a significant moderating role in the influence of job resources and demands on job crafting. Servant leadership can enhance the positive role of job resources and weaken the negative role of job demands. This conclusion is consistent with studies by Ozturk et al. (51) and Bao et al. (52). In our research, we found that those tobacco retailers who rated their leaders highly were generally those with higher levels of work engagement. They proposed that their managers provided professional guidance on their retail work and supported them to take job crafting. Servant leaders took the retailers as the center of work, helped them solve many work problems, motivated the retailers to work harder, and improved their work engagement level.

## Research contributions

### Theoretical implications

First, this study takes tobacco retailers, a flexible group of work forms, as the research object. After the outbreak of the new coronary pneumonia, the isolation and current limiting measures completely break the original human resource management mode. Countermeasures such as telecommuting and flexible employment bring new opportunities and challenges to human resource management. The research on the work engagement mechanism of tobacco retailers' flexible work can also provide some enlightenment for enterprises' flexible employment.

Second, this study constructs the impact path model of job demands and resources on work engagement, enriches the theoretical results of JD–R theory, and takes job crafting as a mediate variable based on self-regulation theory. It reveals that job crafting is an important mediating path for job demands and resources to affect work engagement of tobacco retailers, which further enriches that work characteristics affect the psychological mechanism of work engagement.

Finally, by discussing the mediators of job crafting and of servant leadership, the mechanism and boundary conditions among job resources, job demands, and work engagement are improved. Most of the previous studies have directly focused on the relationship among job resources, job demands and work engagement, but they have not paid attention to the internal mechanism and boundary conditions. On the basis of self-regulation theory, we found that job crafting plays a mediating role on the effects of job resources and demands on job crafting. Taking servant leadership as a moderate variable into the model, we tested the role of leadership style and provided ideas for future research.

### Practical implications

This study provides valuable practical guidance for the tobacco industry. On the one hand, tobacco retailers work outside the company and have a high degree of flexibility, which results in limited job resources available to retailers. On the other hand, the need for tobacco retailers to serve consumers is an emotional labor, which will accelerate resource consumption. Therefore, knowing how to increase the positive role of job resources, reduce the negative aspects of job demands, and ultimately improve work engagement is crucial for tobacco companies.

First, the findings suggest that tobacco companies should increase job resources, which are an important component related to work engagement, and they should optimize job demands, which negatively affect employees' work engagement. Leaders should take a close look at day-to-day job demands and resources. On the basis of specific operational needs, tobacco company leaders should evaluate, revise, improve, and implement specific job resources. Specifically, they can interact with retailers, listen to their needs, understand their tasks, and provide them with feedback. By doing so, leaders can clearly demonstrate their work engagement and present themselves as an important work resource for their employees.

Second, this study confirms that job crafting positively affects work engagement. Therefore, tobacco companies should encourage employees' work crafting behavior and provide training to enhance employees' vocational skills. Studies have shown that organizational performance can be achieved by providing conditions for employees, guiding employees to actively change their work (53, 54). Tobacco company leaders take the initiative to think and create conditions to promote more employees to bring positive changes through job crafting, thereby helping employees produce higher performance. For example, by means of guidance and setting examples, employees are encouraged to propose new suggestions and opinions to stimulate their internal motivation for job remodeling and constantly inject vitality and power into their job crafting behaviors.

Finally, this study confirms the moderating role of servant leadership in the effects of job resources and demands on job crafting and employee work engagement. For a more flexible career of tobacco retailers, the influence of managers' leadership style is particularly obvious. Servant leadership helps every employee in the organization to give full play to their subjective initiative and obtain excellent performance (55, 56). Specifically, tobacco enterprises can strengthen servant leadership characteristics, help them pay attention to the interests and psychological needs of subordinates in practice, and urge subordinates to redesign their work actively to adapt to the effects of job resources and demands and improve the level of work engagement. In the organizational framework of the tobacco company, the account manager assumes the function of servant leadership. Learning and mastering the skills of job crafting interventions can be incorporated into the range of job responsibilities of account managers. Furthermore, companies in other industries cannot ignore informal employees who are away from the organizational face-to-face management but have a significant influence on organizational performance; thus, providing them with servant leaders to guide and reshape their jobs is a smart move.

### Limitations

First, this study used cross-sectional data, and the causal attribution between variables was not sufficiently strict. Future research can use longitudinal research methods and empirical sampling methods to be further effective in exploring the causal relationships between variables (57–59). Second, the data of this study were collected in the form of self-report. Although the common method variance test was conducted to confirm that the common method variance does not have a serious effect on the hypothesis test, the combination of self-report and other evaluation can be considered in future research. Finally, this study took the servant leadership perceived by employees as the moderator variable from the individual level. In the future, variables at the organizational level for cross-level research can be used to further enrich the theory.

## Data availability statement

The original contributions presented in the study are included in the article/supplementary material, further inquiries can be directed to the corresponding author.

## Author contributions

DJ: conceptualization, data, and project management. LN: methodology, formal analysis, and writing-original draft. TL, YZ, and QL: social writing-review and editing. All authors contributed to article and approved the submitted version.

## Conflict of interest

The authors declare that the research was conducted in the absence of any commercial or financial relationships that could be construed as a potential conflict of interest.

## Publisher's note

All claims expressed in this article are solely those of the authors and do not necessarily represent those of their affiliated organizations, or those of the publisher, the editors and the reviewers. Any product that may be evaluated in this article, or claim that may be made by its manufacturer, is not guaranteed or endorsed by the publisher.

## Appendix

**Job Resources [adopted from Demerouti et al.**, **(****9****)]**

(1) Your manager helps and supports you.
(2) You are consulted before objectives are set for your work.
(3) You are involved in improving the work organization or work processes of your department or organization.
(4) You can influence decisions that are important for your work.
(5) I receive the recognition I deserve for my work.
(6) The organization I work for motivates me to give my best job performance.

**Job Demands [adopted from Karasek et al.**, **(****34****)]**

(1) It often seems like I have too much for one person to do.
(2) I have too much work to do everything well.
(3) The amount of work I am asked to do is unfair.
(4) I never seem to have enough time to get everything done.
(5) The amount of time my job takes up makes it difficult to fulfill family responsibilities. e
(6) Things I want to do at home do not get done because of the demands my job puts on me.
(7) My job produces strain that makes it difficult to fulfill family duties.
(8) Due to work-related duties, I have to make changes to my plans for family activities.

**Job Crafting [adopted from Petrou et al**. **(****24****)** **and Demerouti et al**. **(****35****)]**

(1) I ask others for feedback on my job performance.
(2) I ask colleagues for advice.
(3) I try to learn new things at work.
(4) I contact other people from work (e.g., fellow pilots, flight operations manager) to get the necessary information for completing my tasks.
(5) I ask for more responsibilities.
(6) I think of solutions in order to carry out my work more easily.
(7) I look for ways to increase efficiency at work.

**Work Engagement [adopted from** **(****36****)]**

(1) I focus a great deal of attention on my work.
(2) I concentrate a lot on my work.
(3) I pay a lot of attention to my work.

**Servant Leadership [adopted from Liden** **(****32****)]**

(1) When I encounter personal problems rather than work problems, I will also seek help from my superiors.
(2) My superiors emphasized the importance of giving back to the society.
(3) My superiors can quickly find the cause of the problem.
(4) My superiors give me full freedom to solve problems in the best way I think.
(5) My superiors attach great importance to my career development.
(6) My superiors always put my interests before his.
(7) My superiors will not sacrifice moral standards for success.
